# Green Concrete for a Circular Economy: A Review on Sustainability, Durability, and Structural Properties

**DOI:** 10.3390/ma14020351

**Published:** 2021-01-12

**Authors:** Abathar Al-Hamrani, Murat Kucukvar, Wael Alnahhal, Elsadig Mahdi, Nuri C. Onat

**Affiliations:** 1 Department of Civil and Architectural Engineering, Qatar University, Doha 2713, Qatar; aa1205725@student.qu.edu.qa (A.A.-H.); wael.alnahhal@qu.edu.qa (W.A.); 2 Department of Mechanical and Industrial Engineering, College of Engineering, Qatar University, Doha 2713, Qatar; elsadigms@qu.edu.qa; 3 Qatar Transportation and Traffic Safety Center, Qatar University, Doha 2713, Qatar; onat@qu.edu.qa

**Keywords:** green concrete, cement, ground granulated blast furnace slag, fly ash, silica fume, metakaolin

## Abstract

A primary concern of conventional Portland cement concrete (PCC) is associated with the massive amount of global cement and natural coarse aggregates (NCA) consumption, which causes depletion of natural resources on the one hand and ecological problems on the other. As a result, the concept of green concrete (GC), by replacing cement with supplementary cementitious materials (SCMs) such as ground granulated blast furnace slag (GGBFS), fly ash (FA), silica fume (SF), and metakaolin (MK), or replacing NCA with recycled coarse aggregates, can play an essential role in addressing the environmental threat of PCC. Currently, there is a growing body of literature that emphasizes the importance of implementing GC in concrete applications. Therefore, this paper has conducted a systematic literature review through the peer-reviewed literature database Scopus. A total of 114 papers were reviewed that cover the following areas: (1) sustainability benefits of GC, (2) mechanical behavior of GC in terms of compressive strength, (3) durability properties of GC under several environmental exposures, (4) structural performance of GC in large-scale reinforced beams under shear and flexure, and (5) analytical investigation that compares the GC shear capacities of previously tested beams with major design codes and proposed models. Based on this review, the reader will be able to select the optimum replacement level of cement with one of the SCMs to achieve a certain concrete strength range that would suit a certain concrete application. Also, the analysis of durability performance revealed that the addition of SCMs is not recommended in concrete exposed to a higher temperature than 400 °C. Moreover, combining GGBFS with FA in a concrete mix was noticed to be superior to PCC in terms of long-term resistance to sulfate attack. The single most striking observation to emerge from the data comparison of the experimentally tested beams with the available concrete shear design equations is that the beams having up to 70% of FA as a replacement to OPC or up to 100% of RCA as a replacement to NCA were conservatively predicted by the equations of Japan Society of Civil Engineers (JSCE-1997), the American Concrete Institute (ACI 318-19), and the Canadian Standards Association (CSA-A23.3-14).

## 1. Introduction

With the increasing risks of climate change and the depletion of natural resources due to their utilization in the construction industry, sustainability has gained wide importance and the term circular economy (CE) has emerged as one of the most important factors leading to sustainable development [1]. In contrast to the prevailing traditional economy system, which is based on a methodology of make, use, and finally, dispose of, the CE aims at continuous use of products by recycling and reusing instead of disposing to create a closed-loop system and reduce the resource consumption [2].

Evidence suggests that the increasing population growth rate is among the most important factors for urban expansion [3]. Recently, records have shown that compared to 1960, at which the population was only 3 billion, the population dramatically jumped to 7.2 billion in 2017 [4]. This dramatic increase is pressing on the environment, and thus necessitates the allocation of more housing units and service and industrial facilities. As a result, countries are undergoing a revolution in terms of construction to meet the necessary needs.

From a construction perspective, the Portland cement concrete (PCC) is recognized as the most important material that is widely used in different structural applications with abundant raw material. Annually, more than six billion tons of concrete are produced globally, which are equivalent to 1 ton/capita on the planet [5,6]. Previous studies [5,7] reported that in a cubic yard of concrete, 10% by weight contains cement and around 0.9 tons of carbon footprint are generated per 1 ton of cement. In the manufacturing process of cement, two basic raw ingredients, namely calcareous material (i.e., limestone) and an argillaceous material (i.e., clay), are melted at high temperatures of 1400 to 1650 °C, to be transferred to cement clinker [8]. Thus, this process consumes massive amounts of fossil fuels, resulting in a huge carbon footprint [5]. This is beside the carbon footprint induced by the chemical reaction involved to decompose limestone (CaCO_3_) into (CaO + CO_2_) [9,10]. The International Energy Agency (World Energy Outlook 2016) estimated the global carbon footprint to be 21.6 billion tons, of which the cement production accounts for 8% of the total carbon footprint [9,11,12]. Furthermore, in the last decade, the cement industry has become the second-fastest growing industry in releasing CO_2_ emissions due to the growing worldwide demand for concrete [7]. Meanwhile, recent statistics indicated an annual worldwide generation of slag and fly ash (FA) wastes of around 270 to 320 million tons and 1 billion tons, respectively [13,14]. Moreover, in the United States and Norway, the annual output of silica fume (SF) was estimated to be of the order 2 × 10^5^ to 5 × 10^5^ tons [15]. In Turkey, Baspinar and Demir [15] also stated that 700 to 1000 tons of SF were produced from one ferrosilicon production plant. Furthermore, the rice husk ash (RHA) is another highly reactive pozzolanic material obtained as a residue from the pod of rice grains, with a tremendous global amount of 156 million metric tons [16].

Coupled with the cement issue is the tremendous worldwide construction and demolition (C&D) wastes originated from the demolition and reconstruction of old structures, which creates another source of environmental burdens. It has previously been observed that annually, over 500 million tons of C&D wastes are generated worldwide [17]. More recently, Akhtar and Sarmah [18] stated that a global amount of C&D wastes exceeding 3 billion tons are generated annually, where China, India, and the USA are the major contributors to this waste. Subsequently, more land areas are being occupied and polluted when disposing C&D wastes into landfills [19,20]. With this in mind, the global annual consumption of natural coarse aggregates (NCA) has reached 40 billion tons [21], and it is annually increasing by 5%, whereas the highest consumption was concentrated in Asia and the Pacific [22]. This enormous consumption of non-renewable natural resources plays a vital role in depleting natural resources in several countries [23]. As a result, green concrete (GC) has been an object of research since the last century [24]. It is usually referred to as concrete that contributes toward better exploitation of waste materials, less consumption of natural resources, and less carbon footprint [5,25,26,27,28,29,30]. According to Long et al. [31], different strategies were implemented to achieve eco-friendly concrete with improved sustainability. One is reducing the depletion of natural resources by partial replacement of NCA with recycled coarse aggregate (RCA) generated from the C&D wastes. Another approach is by partial substitution of ordinary Portland cement (OPC) with waste supplementary cementitious materials (SCMs), which were categorized according to Liew et al. [32] in three groups: 1—industrial wastes such as ground granulated blast furnace slag (GGBFS), fly ash (FA), and silica fume (SF), 2—agricultural wastes such as RHA, corncob ash (CA), and sawdust ash (SA), and 3—municipal wastes such as glass and plastics. Furthermore, cellulose nanocrystals are other green materials extracted from plants and trees, which when partially substituted by OPC can cause a significant reduction in CO_2_ consumption with improved compressive strength and fracture properties of concrete [33].

## 2. Novelty and Research Objectives

Knowing that PCC production is one of the leading causes of global warming and that there are extensive efforts worldwide to achieve a sustainable environment, this study aims to contribute to the growing research area of GC by conducting a comprehensive review on the sustainability, strength, and durability properties of GC to check for its feasibility as an eco-friendly and structural material instead of the PCC. The GC in this study will be limited to concrete that incorporates RCA as a replacement to the NCA, and either GGBFS, FA, SF, or metakaolin (MK) as a replacement to the OPC. This review paper will allow the user to select the recommended GC constituents that would suit for either low- or high-strength applications by determining the strength ranges either above or below 40 MPa obtained from several studies available in the Scopus database at a certain age, replacement level of cement with one of the SCMs, and water binder (W/b) ratio. Also, this paper will give insights into GC performance in terms of elevated temperature, sulfate attack, chloride ion penetration, and freezing and thawing exposures. Furthermore, this study will analytically illustrate the accuracy of the available design codes and guidelines in predicting the experimental shear capacities of the previously tested GC beams. Therefore, it is intended from this review study to reach for the following:

- Discuss the sustainability benefits of GC on the environment, then provide an overview discussion of the most prominent findings concerned with the mechanical properties of GC in terms of compressive strength.
- Investigate the durability performance of GC under different harsh environmental exposures and then discuss the structural findings on shear and flexural behavior of large-scale reinforced GC beams.
- Collect all shear behavior studies that partially incorporate RCA or SCMs to replace NCA or OPC respectively, and then analytically compare their concrete shear capacities with available design codes and proposed shear equations.

## 3. Literature Review

### 3.1. Review Method

**Figure 1:**
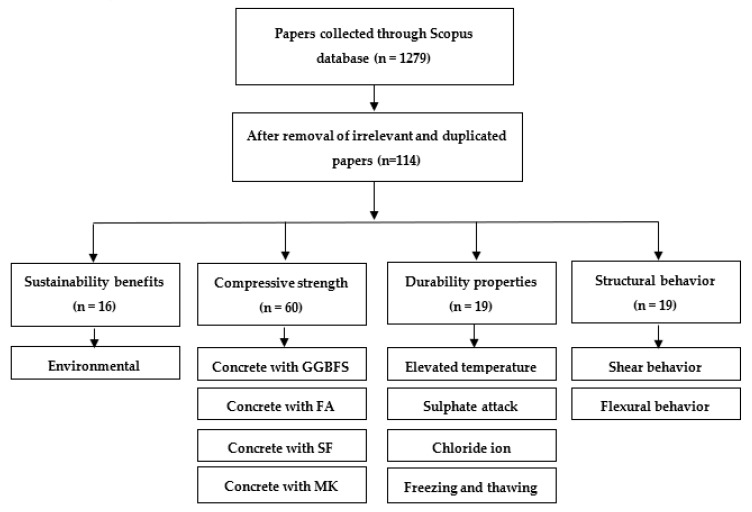
The process used to conduct this review.

The flow chart of the review process is shown in Figure 1. Before commencing the analysis, several research papers were collected through the peer-reviewed literature database Scopus. The total number of collected papers was 1279. The collection was done based on five search categories: (1) GGBFS concrete, (2) FA concrete, (3) SF concrete, (4) MK concrete, and (5) RCA concrete. Under each search category, a block of keywords related to either of four topics was identified, namely, the sustainability benefits, the compressive strength, the durability behavior, and the structural behavior of reinforced GC beams. As shown in Figure 1, the subcategories under these topics referred to the main points that were discussed and reviewed in the paper. The keywords’ block for each search category was specified after refining author keywords or indexed keywords in Scopus. The logic operator “OR” was used to combine the different search terms in each search block. These search blocks were separately searched in the article title, abstract, and keywords search term. Under the compressive strength category, studies were only included in terms of replacing the OPC with SCMs such as GGBFS, FA, SF, and MK, whereas the RCA concrete papers were excluded. However, the RCA concrete papers were included in the remaining categories. Moreover, all non-English, numerical, and fiber-reinforced concrete articles were excluded. Based on this selection criteria and after screening papers’ abstracts to check for relevant research, 114 papers were collected for this review study.

### 3.2. Sustainability Benefits of GC

Worldwide, waste is a growing public health concern. However, recognizing it as a potential source of raw material for the industry would enhance the resource efficiency, because following such a strategy could establish a CE system, by which the materials loops will be closed. Thus, minimizing natural resources depletion, reducing carbon footprint, and eliminating wastes [6,34,35,36]. In the initial phase, the RC’s ingredients are manufactured after supplying the factories with the recommended raw materials, and waste by-products such as GGBFS, FA, and SF to partially replace the OPC and to avoid their disposal into landfills. This is followed by the construction processes and the service life of the building. Whenever needed, the building should be refurbished and repaired to extend its lifetime. At some stage, where the building would reach the end of its life, the demolition action will take place and the generated waste could be recycled for the same or another process.

In the history of sustainable development, the GC has been thought of as a key factor in improving the three sustainability pillars: environmental, economic, and social [25]. This is due to the circularity property found in the GC technique, which will conserve cement and natural resources for NCA, such as shale, limestone, natural rocks, and clay, reduce and save landfill areas and costs, and reduce carbon footprint by reducing the cement demand, which reduces fossil fuels consumption in the cement manufacturing process [34]. Besides, utilizing GC would conserve the water storage capacity of the ground and protect the natural habitat. This is because aggregate deposits act as an underground water reservoir, and when extracted through mining processes, the ground’s storage capacity will be lost. Also, the water drainage patterns will be changed because of the change in the slope of the land and vegetation [34]. Therefore, using an eco-friendly concrete, which utilizes RCA instead of NCA, or utilizes waste SCMs as one of its ingredients to partially replace cement, might have a pivotal role in creating a facility to improve the structural knowledge and maintaining a safe ecological and economical solution. Also, the issue of disposing of these by-products into landfills is a major environmental problem, as they contain a significant amount of leachable toxic elements, which can cause ecological harm to the water, soil, and air [13].

To date, several studies have conducted a comparative life cycle assessment (LCA) between PCC and GC. For example, Knoeri et al. [34] analyzed the LCA of 12 concrete mixes with RCA and found out that the environmental impact was mitigated by 30% compared to their counterpart conventional concretes (CC) with NCA. This mitigation was due to the avoidance of C&D wastes disposal in landfills and the recovered scrap iron from steel reinforcement. This matches well with Yazdanbakhsh et al. [37], where two environmental impact indicators of RCA including the acidification and smog formation were lower than that of NCA by 16% and 17%, respectively. In addition, in their study, Yazdanbakhsh et al. [37] demonstrated a 35% lower environmental impact induced from transporting RCA to the ready-mix plants than transporting NCA. Faleschini and Pellegrino [38] also showed that replacing NCA with electric arc furnace (EAF C) slag in concrete has decreased greenhouse gas emissions by 35%. According to Abbas et al. [39], implementing the RCA in concrete has another advantage of reducing cost, as aggregates are obtained locally rather than being hauled from remote locations. The LCA of Shan et al. [40] was in line with previous findings, where their results have shown a significantly lower environmental load for the local RCA than the NCA imported from overseas. Turk et al. [41] prepared GC mixes from three industrial by-products, which are (1) foundry sand, (2) EAF S (which were used as manufactured aggregates), and (3) FA (which was used as a mineral admixture). Their results indicated a 25% reduction in environmental impacts in the case of FA, 15% in the case of foundry sand, and 5% to 35% in the case of EAF S. Concerning CO_2_ emissions, the case of EAF S showed only minor improvement, while it showed a very big improvement in Eutrophication. Gursel et al. [42] investigated the global warming potential (GWP) of RHA and FA blend concrete mixes through a LCA approach. In comparison to CC, which resulted in a GWP of 544 kg CO_2_-eq/m^3^, it emerged from their analysis that the mix with 40% OPC, 40% FA, 15% RHA, and 5% limestone flour showed the lowest GWP of 284 kg CO_2_-eq/m^3^ without considerable effect on the compressive strength. This finding was also supported by Thomas [43], where an eco-friendly, economical, and durable concrete was presented with the partial replacement of OPC with RHA. While the carbon footprint from normal concrete strength mix was found by Flower and Sanjayan [44] to be 263 to 290 kg CO_2_-eq/m^3^, the replacement of OPC with 25% FA in one mix, and 40% GGBFS in another mix, have shown a 15% and 22% reduction in carbon footprint, respectively. In comparison to cement production, less than a tenth of the carbon footprint is induced from the GGBFS production, with less than a fifth of the energy required to produce cement [45]. In a recent study by Yu et al. [46], the OPC was replaced by not less than 80% of FA targeting a low-strength concrete of 30 MPa. Two material sustainability indicators were adopted in their study focusing only on the manufacturing process of the material used, which were the embodied energy and the embodied carbon content. Interestingly, the GC mix was observed to exhibit 1/4 to 1/3 of both the embodied energy and the embodied carbon footprint of the conventional M30 concrete mix. This environmental improvement was accompanied with a reduced cost by 35% of the M30 mix. A case study by Elchalakani et al. [47] was carried out to prepare an efficient and low carbon footprint concrete mix design to build the city of Masdar in the United Arab Emirates. For this purpose, 13 different concrete mixes with 50% to 80% replacement of OPC with GGBFS were prepared. The test results of concrete mixes made with GGBFS indicated a 60% reduction in the carbon footprint, and therefore, a mix with 80% GGBFS and 20% OPC was nominated for the future construction of Masdar City.

### 3.3. Strength Properties of GC

In this section, the compressive strength properties of GC, which incorporate industrial SCMs such as GGBFS, FA, SF, or MK as one of its ingredients to replace the OPC, will be studied and analyzed. Most of the collected compression tests in this section were done on 100 mm × 200 mm cylinders and a few of the remaining were 150 mm × 300 mm cylinders, 100 mm cubes, and 150 mm cubes.

#### 3.3.1. Concrete with Ground Granulated Blast Furnace Slag (GGBFS)

The slag is a by-product produced during the manufacturing process of steel [48]. It is made up of the same ingredients that make up the OPC, such as alumina, lime, and silica, but with different proportions [49]. As the slag leaves the blast furnace, it must be rapidly chilled to minimize the crystallization of the molten slag and convert it into fine glassy and granulated particles that are smaller than 4.75 mm in size [50]. The granular product is then ground into fine powder to obtain the GGBFS [51].

The results of compressive, flexural, and split tensile strengths for several studies incorporating the GGBFS at different percentages in concrete are presented in Table S1 in the Supplementary File. It has been recorded that compared to the control mixture with 100% OPC, lower compressive strength at 7 days was attained when GGBFS was partially incorporated in concrete [50,52,53,54]. However, the compressive strength of the GGBFS mixture specimens with 25% replacement was higher at 28 days [52]. For 55% replacement of GGBFS, similar and higher compressive strength to that of the control specimen was obtained at 56 and 90 days respectively [52], while the higher compressive strength was obtained at both ages in References [53,55] when using 60% replacement of GGBFS. The optimum level of GGBFS replacement which yields the highest compressive strength was found by Oner and Akyuz [50] to be 55%. Interestingly, Oner and Akyuz [50] noticed that for the same concrete workability, the water binder (W/b) ratio reduces as the GGBFS replacement increases, thus the GGBFS has a positive effect on workability as higher compressive strength can be achieved with lower water consumption. For the flexural strength, Khatib and Hibbert [53] showed that at 90 days of curing, the strength of the 60% GGBFS specimen was enhanced by 19.6% compared to the control specimen. Keeping in mind that the flexural behavior is sensitive to microcracks, the finer particles of GGBFS along with the secondary pozzolanic reaction can reduce the pore connectivity in hardened concrete and as a result, enhance the flexural strength [45,56]. A similar observation was recorded by Guneyisi and Gesoglu [57], where higher compressive and split tensile strengths were achieved at a long time period of 90 days with a replacement level of 60% of GGBFS.

The lower strength of GGBFS concrete at early ages was mainly attributed to the slow pozzolanic reaction of GGBFS, which depends on the calcium hydroxide Ca(OH)_2_ availability forms at later ages [50]. Through the pozzolanic reaction, an extra calcium silicate hydrate (C-S-H) gel will be generated, which will densify the microstructure of concrete, thus higher compressive strength of GGBFS concrete is obtained [55]. To enhance the early strength of GGBFS concrete and for further creation of the (C-S-H) gel, several studies suggested the addition of Ca(OH)_2_ as a hydrated lime [58,59]. Although the early strength of GGBFS concrete was low, this deficiency might be eliminated when adding superplasticizers (SP) at a low W/b ratio. The results for a 20% replacement of GGBFS obtained by Johari et al. [60] revealed higher 7-day compressive strength (79.6 MPa) than the control specimen (74.8 MPa) when 14 Kg/m^3^ of SP was incorporated at a 0.28 W/b ratio. Whereas at 28 and 90 days, comparable and higher strength were obtained at the 60% replacement level.

**Figure 2:**
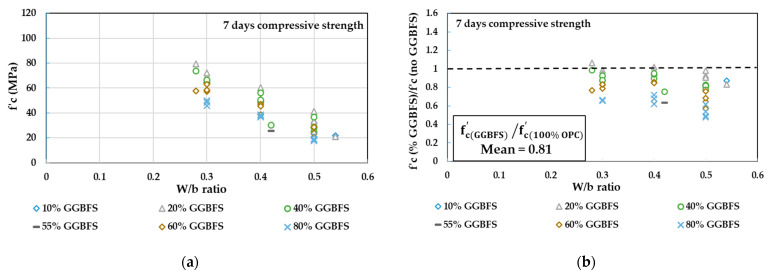

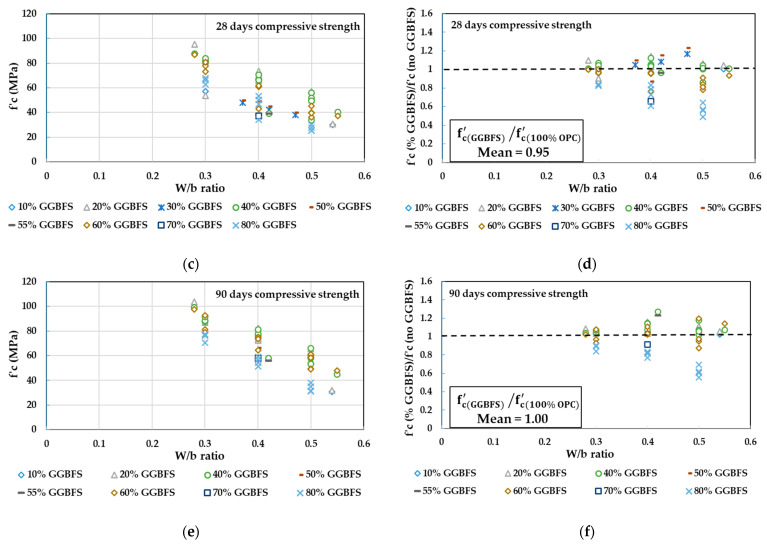
Concrete with Ground Granulated Blast Furnace Slag (GGBFS) as a partially replacing material for cement. Compressive strength vs. water binder (W/b) ratio at (**a**) 7 days; (**c**) 28 days; (**e**) 90 days. Ratio between compressive strength of concrete with GGBFS to concrete without GGBFS at (**b**) 7 days; (**d**) 28 days; (**f**) 90 days.

In Figure 2, the concrete compressive strength values obtained from several tests in the literature [48,50,52,53,55,57,60,61,62,63,64,65] at 7, 28, and 90 days for different replacement levels of GGBFS at different W/b ratios are plotted in Figure 2a,c,e. The ratios between concrete compressive strength at different replacement levels of GGBFS to the reference concrete without GGBFS are also plotted in Figure 2b,d,f to show how close the GGBFS concrete specimens are to the control specimens. The total number of tested specimens is 65. By referring to Figure 2, the following conclusions can be observed:

- At 7 days, the GGBFS addition resulted in a lower strength compared to the concrete without GGBFS, as indicated in Figure 2b. However, similar, or closer strength, was achievable when 20% of GGBFS was added with a W/b ratio ranging from 0.3 to 0.4.
- At 7 days, concrete strength of the range 20 to 35 MPa can be achieved when W/b is in the range of 0.42 to 0.5 and when the replacement ratio of GGBFS is ranging from 20% to 60% (Figure 2a).
- At 7 days, concrete strength of the range 40 to 60 MPa and 60 to 80 MPa can be achieved when W/b is ranging from 0.3 to 0.4 and 0.28 to 0.3 respectively, and when the replacement ratio of GGBFS is ranging from 20% to 60% (Figure 2a).
- The 28- and 90-day tests pointed out more gain in the GGBFS concrete than the control concrete, therefore most of the GGBFS mixtures showed closer lower strength, whereas some were greater than the control specimens. This could reflect the effect of a secondary pozzolanic reaction (Figure 2d,f).
- At 28 days, similar or closer strength to the reference concrete was achievable when 20% to 60% of GGBFS was added with a W/b ratio ranging from 0.3 to 0.42 (Figure 2d).
- At 28 days, concrete strength of the range 20 to 35 MPa can be achieved when W/b = 0.5 with a replacement ratio of GGBFS ranging from 20% to 80% (Figure 2c).
- At 28 days, concrete strength of the range 40 to 60 MPa can be achieved when W/b is of the range 0.3 to 0.4 and when the replacement ratio of GGBFS is ranging from 20% to 80% (Figure 2c).
- At 90 days, the compressive strength for all concrete mixtures with different GGBFS% and W/b ratios exceeded 40 MPa, except for 80% of the GGBFS mixture at a W/b ratio of 0.5 (Figure 2e).
- The ratio of mean GGBFS concrete compressive strength to the 100% OPC concrete compressive strength ($f_{c\left(GGBFS\right)}^{\prime }/f_{c\left(100\%\;OPC\right)}^{\prime }$) was noticed to be closer to 1 as time passed. This reflects the effect of pozzolanic reaction, which develops at later ages by reacting with the hydrated lime to densify the microstructure of concrete, thus, increasing compressive strength.

#### 3.3.2. Concrete with Fly Ash (FA)

The FA is a fine powder by-product resulting as a residue from the burning of pulverized coal at high temperatures in electric generation power plants. It is a pozzolan that comprises mainly silica and alumina, which when mixed with water and lime Ca(OH)_2_ forms a similar compound to Portland cement through the pozzolanic reaction [5], but with a denser and less permeable microstructure [66]. It was reported in the literature that among the worldwide FA production, only 25% was used in the industry [67].

Table S2 in the Supplementary File summarizes the findings of compressive, flexural, and split tensile strengths at different ages, where FA was incorporated in concrete at different percentages. According to Naik et al. [68], the addition of a high percentage of FA (50% to 70%) revealed lower compressive strength than the reference specimen without FA. This observation is consistent with Lam et al. [69], however, comparable strength to the reference was achieved at a lower % of FA (15–25%) at 28 days, while higher strengths at 56 and 90 days were shown. This was limited to the specimens having low W/b = 0.3, whereas lower strength was recorded at higher W/b ratios. Although the results of Bouzoubaa and Lachemi [70] have shown increasing compressive strength with decreasing the FA % from 50% to 40% and W/b ratio from 0.45 to 0.35, the targeted 28-day strength of 35 MPa was attained for all mixtures. In contrast to previous findings, at 50% replacement of cement with FA with W/b = 0.3, Atis and Ash [71] found the compressive strength at 7, 28, and 365 days respectively, to be 48.3, 66.55, and 83.60 MPa compared to the control specimen strengths of 52.63, 64.55, and 77.08 MPa. Han et al. [72] concluded that the addition of 30% of FA has improved the long-term strength at 365 days, while higher early strength at 28 days was obtained when cement was substituted by 10% of FA. Siddique [73] stated that the compressive strength continued to decrease as the replacement ratio increased from 40% to 50% with W/b = 0.4, however, the obtained strengths were sufficient for the use in reinforced concrete structures. It has been demonstrated by Dinakar et al. [74] that for low-strength self-compacting concrete (20 to 30 MPa), the replacement ratio of FA can reach up to 70% to 85%, while for higher strength grades (60 to 90 MPa), the replacement ratio can be in the range of 30% to 50%. In their analysis, Nath and Sarker [66] concluded that when partially replacing cement with fly ash, the 28-day strength will experience a drop if no adjustment to the W/b ratio is applied. Therefore, high-strength concrete of 67 MPa can be obtained at 28 days when adjusting the W/b ratio from 0.41 in the specimen without FA to 0.31 in the specimen with 40% of FA. At 56 days, the strength was remarkably increased to reach 88 MPa, but no further increase in strength was noticed beyond this age. The results obtained by Durán-Herrera et al. [75] draw the attention toward the inefficient use of FA at a replacement ratio exceeding 30% when W/b is equal to or above 0.5, where a significant drop in the strength of 45% was reported at 28 days. Beyond 7 days, the authors of References [76,77] pointed out that the gain in strength for the FA concrete was greater than the reference concrete at 28, 56, 90, and 365 days. By adjusting the W/b ratio, the reference 28-day strength was exceeded at a replacement level of 20% to 40% of FA, but for a higher replacement level of 60% to 80% of FA, the reference strength was exceeded at 90 days [78]. In addition, the FA concrete indicated a superior flexural strength from 28 to 365 days.

The increase in strength when cement was partially replaced with FA is attributed to the re-crystallized calcium carbonate and the creation of additional (C-S-H) gel in the cementitious matrix, which was formed by the interaction between FA and Ca(OH)_2_ that reduces the porosity of both the transition zone and the matrix [79]. Also, the finer particles that fill the voids between aggregates along with their spherical shape will produce a better particle packing and a denser paste, thus the strength will increase [80].

**Figure 3:**
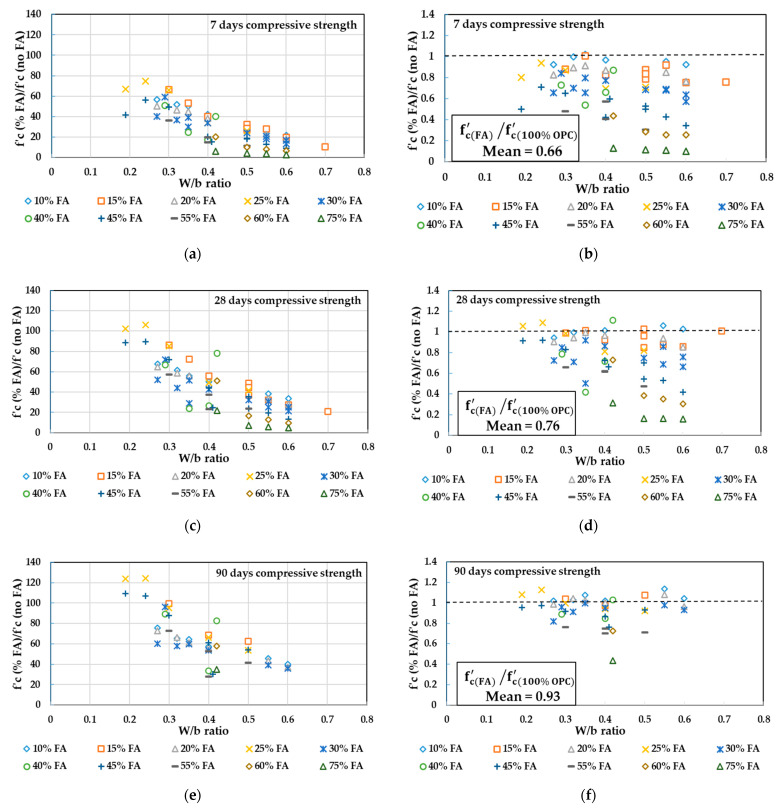
Concrete with Fly ash (FA) as a partially replacing material for cement. Compressive strength vs. W/b ratio at (**a**) 7 days; (**c**) 28 days; (**e**) 90 days. Ratio between compressive strength of concrete with FA to concrete without FA at (**b**) 7 days; (**d**) 28 days; (**f**) 90 days.

In Figure 3, the concrete compressive strength values obtained from the literature [68,69,70,71,72,73,74,75,76,77,79,80,81,82] at 7, 28, and 90 days for different replacement levels of FA at different W/b ratios are plotted in Figure 3a,c,e. The ratios between concrete compressive strength at different replacement levels of FA to the reference concrete without FA are also plotted in Figure 3b,d,f to show how close the FA concrete specimens are to the control specimens. The total number of tested specimens is 63. By referring to Figure 3, the following conclusions can be observed:

- At 7 days, the FA addition resulted in a lower strength compared to the concrete without FA, however similar or closer strength was achievable when 10% to 15% of FA was added with a W/b ratio ranging from 0.3 to 0.4 (Figure 3b).
- At 7 days, concrete strength of the range 20 to 35 MPa can be achieved when W/b is in the range of 0.4 to 0.55 and when the replacement ratio of FA is ranging from 10% to 30% (Figure 3a).
- At 7 days, concrete strength of the range 40 to 60 MPa can be achieved when W/b is ranging from 0.24 to 0.35 and when the replacement ratio of FA is ranging from 10% to 45% (Figure 3a).
- Similar to GGBFS concrete, the 28-day tests of FA concrete were observed to show more gain in strength than the control concrete, therefore most of the FA mixtures showed closer lower strength and few were greater than the control specimens, except for those mixtures having greater than 40% FA and W/b ratio from 0.4 to 0.6, where no evident enhancement in strength was recorded (Figure 3d).
- At 28 days, similar or closer strength to the reference concrete was achievable when 10% to 25% of FA was added with a W/b ratio ranging from 0.24 to 0.35 (Figure 3d).
- At 28 days, concrete strength of the range 20 to 35 MPa can be achieved with W/b of the range 0.5 to 0.6 or 0.24 to 0.45, with a replacement ratio of FA ranging from 10% to 30% or 40% to 60%, respectively (Figure 3c).
- At 28 days, a higher strength grade of the range 40 to 60 MPa and 60 to 80 MPa can be achieved when W/b is of the range 0.27 to 0.4 and 0.24 to 0.36 and when the replacement ratio of FA is ranging from 10% to 55% and 10% to 40%, respectively (Figure 3c).
- At 90 days, the FA mixtures exhibited more gain in strength than the 0% FA mixtures. This could reflect the effect of secondary pozzolanic reaction, which produces a higher rate of strength gaining in the long term (Figure 3f).
- The ratio of mean ($f_{c\left(FA\right)}^{\prime }/f_{c\left(100\%\;OPC\right)}^{\prime }$) was recorded as 0.66 at 7 days and it keeps increasing up to 0.93 at 90 days. Although this reflects the effect of pozzolanic reaction as in the case of GGBFS, the strength is developing at a slower rate.

#### 3.3.3. Concrete with Silica Fume (SF)

The SF is another efficient pozzolan with a highly fragmented structure, that when used in the concrete, reacts with the lime produced from the hydrated cement to reduce the pore size volume and capillaries in the cement paste [83]. SF is a waste product produced in the metallurgical industry from silicon alloys such as ferrosilicon, metallic silicon, etc. [84]. Its tiny particles are characterized by microscopic spherical shape with a diameter ranging from 0.1 to 0.5 micrometers (μm) [85].

In the Supplementary File, Table S3 lists the compressive, flexural, and split tensile strength results at different ages for several studies incorporating the SF at different percentages in concrete. The most prominent outcome to emerge from Table S3 is the higher early compressive strength of SF concrete than the reference concrete at 7 days [83,86,87,88,89,90,91]. The compressive strength continues to increase significantly up to 56 days, however, only a marginal increase was recorded beyond this age [86,87,88]. The flexural strength was also enhanced upon the SF addition, and the optimum amount of SF was found to be 15%. This is in complete agreement with reference [90]. Although exceeding this limit decreases the strength, high-strength concrete of 77.5 MPa was still achievable at 25% of SF with a W/b ratio of 0.3 and SP of 12.6 kg/m^3^ [86]. Wong and Razak [88] prepared several concrete mixes having 0% to 15% by weight of cement as SF with different W/b ratios of 0.27, 0.3, and 0.33. Their results observed no immediate enhancement in strength at 3 days due to the SF addition, but from 7 days onward, higher strength than the control concrete was obtained at all ages until reaching 17% increment at 90 days for 10% SF concrete. This could be referred to the slow nature of pozzolanic activity at early ages and the dilution effect of pozzolan. It was also noticed that reducing W/b ratio from 0.3 to 0.27 did not excite a significant increase in strength as expected. In their research, Bhanja and Sengupta [92] have also studied the effect of several W/b ratios, namely 0.27, 0.3, 0.38, and 0.42 on concrete compressive, flexural, and tensile strengths with the SF incorporation at 0% to 30% by weight of cement. It emerged from their results that the optimum replacement level of SF for tensile strength was a function of the W/b ratio in the mix, which confirms the previous finding [70]. The optimum replacement level for tensile strength at 28 days was found to be in the range of 5% to 10%, while for compressive and flexural strengths, it was found to be in the range of 15% to 25%. In comparison to split tensile strength, the flexural strength demonstrated greater improvement due to SF incorporation.

From the previous findings of GGBFS and FA, the 7- and 28-day strengths were reduced compared to the control specimens without GGBFS or FA, whereas comparable or higher strengths were achieved at later ages of 56 and 90 days. In contrast, the early age strengths at and after 7 days have shown a clear enhancement over the control concrete when cement was partially replaced with SF. This was attributed to the smaller size particle of SF than the GGBFS and FA, which leads to an increase in the pozzolanic reaction between SiO_2_ from SF and Ca(OH)_2_ resulting from the hydration of cement [93,94,95,96], which generates a C-S-H gel that grows into the capillary voids of the mortar, thus forming a denser microstructure. Furthermore, the physical role of SF as a filler also aids in the strength development, as the fine particles of SF would lead to a reduction in porosity of the transition zone, and hence the interlocking mechanism between the paste and aggregate is boosted [89,97].

**Figure 4:**
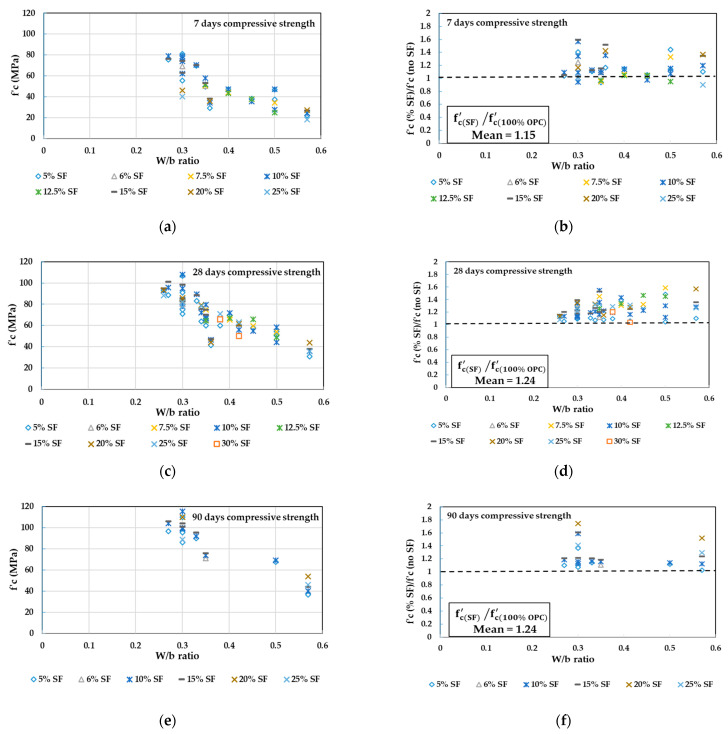
Concrete with Silica fume (SF) as a partially replacing material for cement. Compressive strength vs. W/b ratio at (**a**) 7 days; (**c**) 28 days; (**e**) 90 days. Ratio between compressive strength of concrete with SF to concrete without SF at (**b**) 7 days; (**d**) 28 days; (**f**) 90 days.

In Figure 4, the concrete compressive strength values obtained from the literature [83,86,87,88,89,90,91,92,98,99] at 7, 28, and 90 days for different replacement levels of SF at different W/b ratios are plotted in Figure 4a,c,e. The ratios between concrete compressive strength at different replacement levels of SF to the reference concrete without SF are also plotted in Figure 4b,d,f to show how close the FA concrete specimens are to the control specimens. The total number of tested specimens is 78. By referring to Figure 4, the following conclusions can be observed:

- Unlike GGBFS and FA, the SF addition resulted in approximately a similar and, in most cases, a higher compressive strength compared to the concrete without SF at 7 days (Figure 4b).
- At 7 days, concrete strength of the range 20 to 35 MPa can be achieved when W/b is in the range of 0.36 to 0.57 and when the replacement ratio of SF is ranging from 5% to 20% (Figure 4a).
- At 7 days, concrete strength of the range 40 to 60 MPa can be achieved when W/b is ranging from 0.3 to 0.5 and when the replacement ratio of SF is ranging from 5% to 15% (Figure 4a).
- The 28-day tests resulted in a higher gain in strength in the SF concrete than the control concrete. Therefore, all the SF mixtures showed greater strength than the control specimens (Figure 4d).
- At 28 days, most of the compressive strength values were >40 MPa. High-strength grades of the range 40 to 60 MPa and 60 to 90 MPa can be achieved when W/b is of the range 0.35 to 0.5 and 0.26 to 0.4 and when the replacement ratio of SF is ranging from 5% to 20% and 5% to 25%, respectively (Figure 4c).
- At 90 days, the SF mixtures continue to increase in strength beyond 100 MPa for 10% to 20% of SF concrete with a W/b ratio of 0.27 to 0.3 (Figure 4e).
- The ratio of mean ($f_{c\left(SF\right)}^{\prime }/f_{c\left(100\%\;OPC\right)}^{\prime }$) was reported as 1.14 at 7 days, then it was increased up to 1.24 at 28 days, but at 90 days, the mean ratio remained as 1.24. This indicates the fast and minor strength development at early and later ages, respectively.

#### 3.3.4. Concrete with Metakaolin (MK)

Unlike GGBFS, FA, and SF, the MK is not a by-product, but it is made by the calcination of high-purity kaolin clay at a temperature ranging from 650 to 800 °C [100]. The exposure of the kaolin clay to this range of temperature is done to break down the crystalline structure and remove the chemically bound water from the interstices of the kaolin so that the material is converted into an amorphous aluminosilicate called MK [91]. During its manufacturing, the MK passes through a well-controlled process that carefully refines the particles to drive off the inert impurities, lighten its color, and results in a high reactivity powder with high consistency in performance and structure [91]. In comparison to a cement particle size of 10 μm, the MK has a median particle size of 1.3 μm [101,102].

Different studies that partially substituted the OPC with MK are provided in Table S4 of the Supplementary File. Zhang and Malhotra [101] reported that the compressive strength of 10% MK concrete has exhibited higher compressive strength values than the control concrete at all ages up to 180 days. This observation was further supported by References [103,104,105,106] and when compared to SF, the MK showed a faster increment in strength at the early ages of 3 days, which also concurs well with references [98,102]. At a higher replacement level of 20% MK, Khatib and Hibbert [53] outlined that no further enhancement in strength was recorded. Also, Khatib and Hibbert [53] concluded that the replacement level of 10% MK was the best, and it was found to be superior to SF in terms of strength development, particularly at an early age of 3 days, where higher strength than the control was triggered, while for SF, higher strength than control was triggered at or after 7 days. Dinakar et al. [107] indicated that at an optimum replacement level of 10% MK, a strength value of 100 MPa can be obtained at a low W/b ratio of 0.3. The same concrete mix has resulted in 28 days splitting tensile strength of 5.15% of its compressive strength with a relatively high elastic modulus. Ramezanianpour and Jovein [108] stated that the gaining level of compressive strength was developed at lower W/b and with the increasing curing period of concrete. In their study, the optimum amount of MK for concrete with a W/b ratio of 0.35 and 0.4 were 10% and 12.5%, respectively. However, according to the literature, the optimum amount of MK for 40 to 50 MPa concrete at a 0.5 W/b ratio was found to be 20% [53,102,109,110,111], whereas it was found to be 10% for 80 to 100 MPa concrete at W/b of 0.3 [28,88,98,101,105]. The fast strength development of MK in concrete was mainly attributed to the pore filling effect and the fast pozzolanic reaction of MK with the liberated Ca(OH)_2_ during cement hydration, which creates more bonds among the densely packed particles through the formation of C-S-H gel [112]. Moreover, this could also be attributed to a higher content of aluminum oxide (Al_2_O_3_), which caused much higher pozzolanic activity [113].

**Figure 5:**
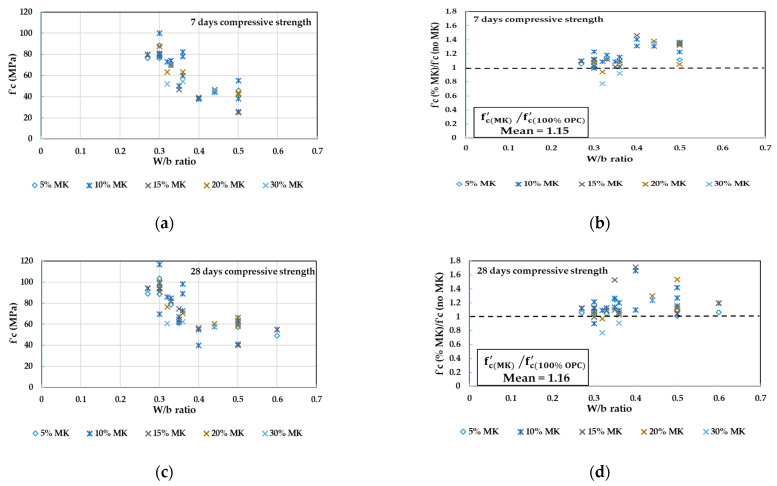

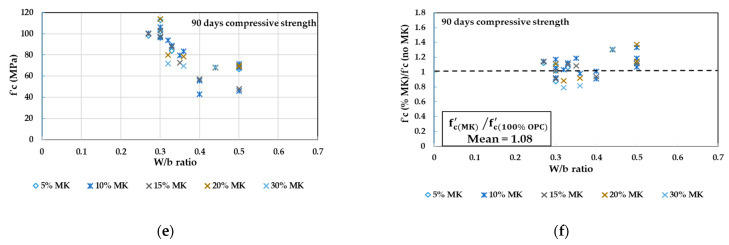
Concrete with Metakaolin (MK) as a partially replacing material for cement. Compressive strength vs. W/b ratio at (**a**) 7 days; (**c**) 28 days; (**e**) 90 days. Ratio between compressive strength of concrete with MK to concrete without MK at (**b**) 7 days; (**d**) 28 days; (**f**) 90 days.

In Figure 5, the concrete compressive strength values obtained from the literature [53,74,88,91,98,101,102,103,106,108] at 7, 28, and 90 days for different replacement levels of MK at different W/b ratios are plotted in Figure 5a,c,e. The ratios between concrete compressive strength at different replacement levels of MK to the reference concrete without MK are also plotted in Figure 5b,d,f to show how close the MK concrete specimens are to the control specimens. The total number of tested specimens is 51. By referring to Figure 5, the following conclusions can be observed:

- Similar to SF, the MK addition revealed higher early compressive strength than the concrete without MK at 7 days. However, lower strength was obtained when replacing cement by 30% of MK at W/b of 0.32 to 0.36 (Figure 5b).
- At 7 days, most of the compressive strength values were >40 MPa. This clearly illustrates the high efficiency of MK in attaining high-strength values at early ages (Figure 5b).
- At 7 days, concrete strength of the range 40 to 60 MPa can be achieved when W/b is ranging from 0.32 to 0.5 and when the replacement ratio of MK is ranging from 10% to 30% (Figure 5a).
- The 28-day tests have shown more strength gain in the MK concrete than at 7 days. High strength grades of the range 60 to 80 MPa can be achieved when W/b is of the range 0.3 to 0.36 and when the replacement ratio of MK is ranging from 10% to 20% (Figure 5c).
- At 28 days, high strength grades of the range 80 to 100 MPa can be achieved when W/b is of the range 0.27 to 0.33 and when the replacement ratio of MK is 5% to 15%, respectively (Figure 5c).
- At 90 days, the MK mixtures continue to increase in strength beyond 100 MPa for 5% to 20% of MK with a W/b ratio of 0.27 to 0.3 (Figure 5e).
- Even at 28 (Figure 5d) and 90 days (Figure 5f), when replacing cement by 30% of MK at W/b of 0.32 to 0.36, lower strength than the reference was shown, whereas higher strength was achieved at a higher W/b ratio of 0.44.
- Based on the ratios of mean ($f_{c\left(MK\right)}^{\prime }/f_{c\left(100\%\;OPC\right)}^{\prime }$) which were reported as 1.15, 1.16, and 1.08 at 7, 28, and 90 days respectively, the MK is very effective in gaining higher early strength at 7 days than the 100% OPC concrete, but this effect turned out to be marginal at later ages of 90 days.

### 3.4. Durability Performance of GC

Durability is one of the most frequently stated concerns with concrete as the deterioration of RC elements could be related, at most, to the harsh environmental exposures [65]. Hence, several durability studies will be presented in this section to discuss the durability performance of GC.

#### 3.4.1. Elevated Temperature

Poon et al. [114] evaluated the effect of elevated temperature up to 800 °C on the performance of eight normal and high-strength concrete mixes, where MK replaced OPC at 0%, 5%, 10%, and 20%. To achieve high-temperature exposure, the test specimens were placed in an automatic electric furnace. Compared to concretes with OPC, FA, and SF, the MK concrete mixes attained higher compressive strength up to 400 °C, whereas beyond 400 °C, a sharp reduction in compressive strength was attained, followed by severe cracking and explosive spalling, which is attributed to its dense micro-structure that allows the build-up of pore pressure by steam [115]. However, the concrete mix with 5% of MK showed better performance than the corresponding concretes at all temperatures without spalling at failure [114].

The mechanical behavior of concrete, where the OPC was replaced by weight with 20%, 40%, and 60% of GGBFS and exposed to temperatures up to 350 °C has been explored in Reference [116]. It was pointed out in the authors’ analysis that the deterioration in compressive strength, splitting tensile strength, and elastic modulus of GGBFS concrete at all elevated temperatures (100, 200, and 350 °C) remained below 40% at 28 and 56 days compared to the mix at room temperature of 27 °C. Among all GGBFS mixes, the 20% GGBFS mix provided the best performance and it could be suitably implemented in nuclear structures.

Li et al. [117] also utilizes GGBFS in concrete with replacement ratios of 10%, 30%, and 50% by weight of OPC to evaluate their performance under high-temperature exposures from 150 to 700 °C for 90 days. The mixes with a higher content of GGBFS have shown higher carbonation depth, and in comparison to the control mix with 100% OPC, the depth was measured as twice as great in the GGBFS concrete when the temperature was raised above 300 °C. The compressive strength was decreased with increased temperature, and this was more pronounced at temperatures higher than 400 °C. As an example, the reductions in compressive strengths for concrete with 0%, 10%, 30%, and 50% of GGBFS were measured at 500 °C as 40%, 38%, 56%, and 59% respectively, compared to the unheated specimens. Moreover, the deterioration in the elastic modulus of GGBFS concretes was more severe than the unheated specimens with percent retentions of 22%, 25%, and 27% respectively, for concretes with 10%, 30%, and 50% of GGBFS.

The mechanical and durability performances of high-performance concrete mixes with 5% to 20% of MK and 20% to 60% FA were also studied under elevated temperatures in Reference [118]. The concrete mixes were exposed to temperature values ranging from 27 to 800 °C followed by slow cooling in air or fast cooling in water. Generally, it was observed that the exposure to 400 °C followed by fast cooling caused more severe degradation in compressive strength. From a durability perspective, the values of sorptivity and chloride permeability were significantly increased for all mixes between 400 to 600 °C due to the increased pore area fraction at a higher temperature. However, at normal temperature, the MK specimens demonstrated higher resistance against water penetration than the FA and CC specimens. On the other hand, the lowest sorptivity was attained for the 20% FA mix at 600 °C and above.

Recently, Rashad and Sadek [113] attempted to improve the compressive strength of 70% GGBFS paste exposed to elevated temperatures, namely 400, 600, 800, and 1000 °C for 2 h. The authors suggested the addition of 2% to 10% of MK as a replacement ratio for the GGBFS by weight. Their results have shown that the compressive strength was enhanced with the increased content of MK before and after the exposure to elevated temperatures. At 800 to 1000 °C, the residual compressive strength for pastes with 2%, 4%, 6%, 8%, and 10% of MK were 10%, 15%, 20%, 27%, and 35% higher respectively, than the control mix with 0% MK.

#### 3.4.2. Sulfate Attack, Chloride Ion Penetration, and Freezing and Thawing

Li and Zhao [119] assessed the short- and long-term resistance to sulfate attack of three concrete mixes, namely CC, concrete with 40% FA, and concrete with a combination of 25% FA and 15% GGBFS (GGFAC). The test was carried following the Chinese Standard GBJ82-85 by immersing specimens with a size of 100 mm × 100 mm × 300 mm in a solution with 2% of H_2_SO_4_ at room temperature. After 50 weeks of exposure, the GGFAC was superior to CC and 40% FA concrete in terms of sulfate attack resistance. Moreover, the change in weight of GGFAC was slow and remained below 8%. This was followed by 10% in the 40% FA mix, while in CC, the weight change reached as much as 16%.

McCarthy and Dhir [120] carried out durability-related tests including chloride diffusion, permeability, and absorption for concrete with 45% FA as a cement component. The chloride diffusion test was done on a concrete cylinder slice of 100 mm diameter and 25 mm depth. The sliced concrete was placed between saturated 5 M NaCl and Ca(OH)_2_ solutions at 20 °C. Whereas, the permeability test was applied on a concrete core of 54 mm diameter × 50 mm depth by recording the flow rates of air passing through the specimen at various inlet pressures. The water absorption of concrete was measured according to BS 1881: Part 208 [121] by immersing a 150 mm concrete cube in a 200 mm head of water for 10 min. In their analysis, McCarthy and Dhir [120] found an enhanced durability performance of FA concrete over the CC in all tests, but for carbonation depth, the performance of FA concrete was similar to that of CC, although, at low design strength, the FA concrete could result in more unsatisfactory performance.

Hossain and Lachemi [122] investigated the suitability of high content of volcanic ash (VA) up to 75% on the strength and durability properties and noticed that compared to the control mix, the drying shrinkage (DS) of VA mixes was slightly lower, however, all mixes experienced less than 600 micro-strains of DS. Moreover, increasing the VA content up to 40% showed a decreased 91-day permeability from 2.23 × 10^−10^ to 1.58 × 10^−10^ cm/s. Also, all VA mixes recorded a chloride ion resistance of 1000 to 3000 Coulombs, which according to ASTM C1202 [123] were classified as low to moderate chloride ion penetrability. The mix with VA beyond 40% was not recommended as it caused a sharp drop in compressive strength.

Kim et al. [124] investigated the durability of concrete while incorporating 0%, 5%, 10%,15%, and 20% of MK and SF. Properties such as chloride ion permeability was reduced as the proportions of MK and SF were increased. Up to 300 cycles of freezing and thawing applied as per ASTM C666 [125], the relative dynamic elastic modulus of concrete mixes with 0% to 10% MK or SF remain constant. The resistance of concrete to carbonation was assessed by subjecting concrete to accelerated conditions involves 5% CO_2_, 30 °C, and 60% relative humidity for 7, 14, 28, and 56 days. Regardless of admixture type (MK or SF) in concrete, the carbonation depth was higher than that of the control mix (with no FA and SF) at all ages of the test from 7 to 56 days. When assessing the sulfuric acid attack of 2% acid solution for 56 days, the authors found a 20% reduction in compressive strength of mortar specimens with 15% MK or SF compared to the control mix. 

Hossain and Lachemi [126] replaced the OPC by 5%, 10%, 15%, and 20% of VA to assess concrete’s durability. Their analysis demonstrated higher resistance of all VA mixes against chloride diffusion than the control concrete with 0% of VA. This observation was also confirmed by performing differential scanning calorimetry tests, which revealed less $\mathrm{Ca}{\left(\mathrm{OH}\right)}_{2}$ content in all VA mixes than the control mix. This indicated that the $\mathrm{Ca}{\left(\mathrm{OH}\right)}_{2}$ was consumed due to the pozzolanic reaction and as a result, created a denser microstructure with very low permeability.

Berndt [127] studied the effect of combining the partial replacement of OPC and NCA with SCM and RCA, respectively. In their results, concrete mix with either NCA or RCA was best performed in terms of mechanical and durability behavior when 50% of cement was replaced with GGBFS. Also, the presence of GGBFS in recycled concrete has decreased the coefficient of chloride diffusion, however, this coefficient along with the permeability coefficient was increased when FA and RCA were employed. In a similar investigation by Kou and Poon [128], concrete mixes with 0%, 50%, and 100% of RCA were prepared. In these mixes, the authors also incorporated FA at different percentages of 25%, 35%, and 55% to evaluate their long-term (10 years) performance in terms of mechanical and durability characteristics. During this period, the concrete specimens were either cured by water or air. The control mixes with NCA have shown higher compressive strength than the recycled concretes at all ages, but this difference was noticed to decrease with the increase in the curing time. Although the recycled concrete had a more permeable structure than the control specimens, the incorporation of FA has led to a significant enhancement in the chloride ion penetration resistance. As the RCA and FA contents were increased, the carbonation coefficient increased. In general, the authors concluded that the optimal concrete mix was that with 50% RCA and 25% FA. In a more recent study, Faella et al. [129] combined the use of RCA with FA in concrete to investigate its durability performance and found that although the addition of RCA induced lower resistance to chloride-ion penetration due to high porosity of RCA, the addition of FA can achieve a significant attenuation of this phenomena.

Sabet et al. [130] measured the effect of FA and SF on the chloride permeability, electric resistivity, and water absorption of concrete. After 90 days of exposure to sodium chloride (NaCl) solution, their analysis showed that the incorporation of 10% and 20% FA caused a reduction in the chloride diffusion coefficient from 7.9 × 10^−12^ to 4.7 × 10^−12^ and 3.2 × 10^−12^ m/s^2^, respectively. For concrete with 10% SF, the chloride diffusion coefficient was reduced to 5.6 × 10^−12^ m/s^2^. In addition, the 10% and 20% FA concrete enhanced the electrical resistivity from 8.4 kΩ cm in the control mix to 30 and 50 kΩ cm, respectively. However, the 10% and 20% SF concrete resulted in the most significant enhancement with 54 and 231 kΩ cm respectively, where kΩ refers to kilo-ohms. Moreover, the final absorption of water was reduced by 20% and 39% when 10% of FA and 10% of SF was incorporated in concrete, respectively.

Chousidis et al. [131] employed lower replacement levels of FA, namely, 5% and 10%, to partially replace the OPC in RC specimens. These specimens were immersed for 130 days in a 3.5% NaCl solution to investigate their mechanical and durability characteristics. In terms of durability, the FA mixes’ sorptivity and capillary absorption were decreased in comparison with the 100% OPC concrete. Moreover, the mass loss of steel reinforcement embedded in 5% FA mortar was measured theoretically to be equal to that of the control concrete after 13 months of exposure to NaCl. In terms of mechanical properties, the compressive strength and elastic modulus at 100 and 130 days were higher in the FA mixes due to the increased density caused by the formation of additional C-S-H.

Singh et al. [132] examined the durability effect of 3% incorporation of silica nanoparticles (SNPs) into concrete mixes having 30% to 50% FA. The main durability parameters were the sulfate attack and the carbonation depth, both were applied for 28, 90, and 180 days. The sulfate attack test was carried out according to ASTM C1012 [133] by slicing prisms of size 100 mm × 100 mm × 500 mm into 50 mm × 100 mm × 100 mm and immersing them inside a solution with 5% magnesium sulfate. The carbonation depth test was applied in accordance with the recommendations of RILEM CPC-18 [134] on specimen size of 100 mm × 100 mm × 500 mm. The specimens were placed in a 2% CO_2_ concentration chamber with 65% relative humidity and a temperature of 20 °C. In comparison to concrete with 30% of FA, the incorporation of 3% of SNPs in a 30% FA concrete has reduced the carbonation depth and the sulfate attack by 73% and 39% respectively, while a 35% and a 30% reduction was observed with the incorporation of 6% SF, respectively.

Wang et al. [135] investigated the durability characteristics of concrete, containing SF at 5%, 8%, and 11%, and FA at 10%, 15%, and 25% by weight to replace the OPC, under the combined effect of sulfate attack and freezing–thawing cycles. Prismatic concrete specimens with a size of 100 mm × 100 mm × 400 mm were immersed in 5% and 10% sodium sulfate solutions and then exposed to 175 freezing–thawing cycles. Conforming to ASTM C666 [125], one freezing and thawing cycle involved 6 h, 3 h for freezing at −18 ± 2 °C, and 3 h for thawing at 5 ± 2 °C in water. The test results indicated significant improvement in concrete durability for concrete with FA up to 25% and 5% to 8% SF. More freezing and thawing cycles (300 cycles) were applied by Uysal and Akyuncu [136] on concrete mixes having 10% to 17% FA as a replacement to the OPC. The results indicated no dramatic change in the weight of specimens, however, the weight loss in FA mixtures was greater than that in the control mix with no FA. The authors also observed a 5.38% to 29.83% loss in the flexural strength of specimens containing FA compared to the control specimens with 100% OPC.

### 3.5. Structural Performance of GC in Large-Scale RC Beams

#### 3.5.1. Partially Replacing OPC with SCM

As discussed in the previous sections, the compressive strength of GC has been analyzed by many studies; however, only a limited number of studies were published on its structural behavior. For example, Yoo et al. [137] evaluated the effect of high volume FA (HVFA) with a 35% and 50% replacement ratio of OPC on the flexural behavior of RC beams. Their results have shown a quasi-similar behavior to the RC members without FA in terms of cracking load, ultimate load, yielding load, and strain, however, results have shown slightly lower elastic modulus and higher mid-span deflection in HVFA beams than the control beams without FA. This could be justified by the known 25% lower density of FA than the cement material, which results in a 2 to 3% reduction in concrete’s unit weight. A similar observation was noticed by Hashmi et al. [138] where the ultimate and yielding state of RC beams with 60% FA were identical to the RC beams without FA, but the RC beams with FA have demonstrated higher deflection and strain values which were attributed to the lower elastic modulus and splitting tensile strength of FA concrete. Sangeetha and Joanna [139] observed that the moment capacity in RC beams with a 40% replacement ratio of GGBFS was comparable to that of the control RC beams without GGBFS at 28 days, however, interestingly it was increased by 21% at 56 days. This could be justified by the enhanced durability [139,140,141] and corrosion resistance [142,143,144] resulted from the fine glassy shape particles of GGBFS, which reduces chloride-ion permeability, and increases the bond between particles [145,146,147,148,149]. Also, Sangeetha and Joanna [139] reported that the crack width at service loads was found to be in the range of 0.17 to 0.2 mm, which is within the limits specified by IS 456-2000 [150]. A more recent study by Hawileh et al. [151] involved a higher replacement level of GGBFS by 70% and 90%. Their results have shown a reduction in the strength and stiffness of beams with 90% of GGBFS by 6% and 16%, respectively, but those beams with 50% and 70% of GGBFS were found practical and increased the ultimate load capacity by 3% and 9%, respectively. Although 90% of GGBFS had sacrifices the flexural strength and stiffness to a small degree, it has increased the RC beams ductility. 

On the other hand, the shear behavior of RC beams when 50% of OPC was replaced by FA was studied by Rao et al. [152]. Their experimental findings have shown a slightly lower shear strength of the FA beams than the conventional concrete (CC) beams. On the contrary, Arezoumandi and Volz [153] tested 12 full-scale beams with two FA contents by weight (50% and 70%) and stated that the FA beams were virtually identical to the CC beams in terms of cracking load, load-deflection diagram, and failure mode, however, beams with FA were noticed to exceed the code-predicted shear capacities by a higher margin than the beams without FA. This could be attributed to the higher fracture energy formed in the cementitious matrix of FA than the conventional OPC. Alghazali and Myers [142] investigated the shear behavior of large-scale beams with three replacement levels of FA by weight (50%, 60%, and 70%) and two different longitudinal reinforcement ratios ρ (1.59% and 2.71%). The FA beams exhibited higher shear strength than the CC ones at a lower ρ of 1.59%, whereas no obvious increase in the ultimate shear capacity was observed at higher ρ of 2.71% but the diagonal shear crack propagation was delayed between 10 to 24%. This observation is referred to the use of a small aggregate size of 10 mm which decreases the crack surface’s roughness and minimizes the effect of the longitudinal reinforcement to prevent slippage.

#### 3.5.2. Partially Replacing NCA with RCA

Central to the entire discipline of sustainable construction is the concept of utilizing RCA in RC structures. In China, the RCA concrete was successfully implemented at various pavements and building structures [154]. Hoffmann et al. [155] have also highlighted that the RCA was suitably used in a wide variety of reinforced concrete members. Numerous studies have attempted to relate the inferior properties of RCA concrete to the weak interfacial transition zone between the recycled aggregate and the new cement paste [156,157,158,159,160,161,162,163], which is mainly attributed to the old layer of mortar adhered to the surface of aggregate [164] that is characterized by loose, porous and micro-cracked surface [159,165]. In their study Han et al. [166] stated that the RC beams with 100% RCA showed larger deflection and less shear strength than the control beams with virgin aggregate. However, Al Mahmoud [167] reported similar shear behavior of the RCA beams compared to the NCA beams in terms of the load-deflection diagram. González-Fonteboa and Martínez-Abella [168] have investigated the shear behavior of recycled concrete with 50% RCA and highlighted little differences in terms of midspan deflection and ultimate load capacity. However, notable splitting cracks and premature cracking were observed along the tension reinforcement of the recycled concrete beams. Etxeberria et al. [169] explored the possibility of implementing the RCA as a structural material in RC beams by replacing the virgin aggregates by 25%, 50%, and 100% of RCA. The beam specimens with 50% and 100% of RCA demonstrated similar shear capacity as the control beams with 0% of RCA, but a reduction of 13% was observed for beams with 25% of RCA. Also, it was noticed that the addition of RCA has reduced the cracking load due to the occurrence of cracking at the weakest point which is the adhered mortar on the surface of RCA. Fathifazl et al. [170] observed that the shear capacity of recycled RC beams with 64% and 74% replacement level of RCA tended to increase at lower shear span to depth ($a_{s}/d$) ratio as a result of the arch action mechanism. Furthermore, Fathifazl et al. [170] observed that the shear capacity tended to increase when the overall depth of the beam was decreased. These two observations indicated that the recycled RC beams conformed well to the known behavior of conventional RC beams. Knaack and Kurama [171] prepared two types of recycled concrete mixes, one with 50% of RCA, and one with 100% of RCA. These mixes were utilized in full-scale RC beams to investigate their flexural and shear behavior. The tested beams exhibited lower initial stiffness and higher ultimate flexural deflection as the RCA replacement level was increased, whereas a relatively small change in the shear and flexural strength was noticed in comparison to the conventional beams with NCA. Arezoumandi et al. [172] undertaken an experimental work that investigates the shear behavior of RC beams where the NCA was totally replaced with RCA. What was emerged from this study is that the beams with 100% RCA were virtually identical to the CC beams in terms of load deflection response, crack progression, and crack morphology. 

### 3.6. Comparing the Experimental Shear Capacities with Major Design Code Provisions 

The available RC design codes; in the design process of an RC beam; specify certain limits on the flexural reinforcement quantity to resist the applied moment first, and to ensure a gradual flexural failure second. However, this is not the case for failure under shear, which usually behaves in a brittle manner with little or without prior warning. Consequently, it is important to analytically investigate the ability of major RC design codes in predicting the experimental concrete shear capacity ($\;V_{c}$) of RC beams; where NCA and OPC are replaced with RCA and SCMs, respectively.

In this section, the concrete shear capacity will be evaluated according to several design codes namely, AASHTO LRFD-2012 [173], ACI 318-19 [174], CSA-A23.3-14 [175], and JSCE-1997 [176] and a proposed equation by Xu et al. [177]. The simplified shear design methods are listed in Table 1. For analysis, two sets of beams were collected from the available literature [141,142,166,169,170,171,172,178,179,180,181,182]. The first set consisted of 35 RC beams, 28 of whom the OPC was partially replaced with SCM, while in the remaining 7 RC beams the OPC and the NCA were both replaced with SCM and RCA, respectively as can be noticed in Table 2. The second set consisted of 41 beams on which the NCA only was partially replaced with RCA (Table 3).

In reviewing the literature, no data was found on the shear behavior of RC beams with GGBFS, SF, or MK as a cement component. On the other hand, several studies have investigated the shear behavior of large-scale RC beams while utilizing FA as a partially replacing material to the OPC, as summarized in Table 2.

Before analyzing the prediction accuracy of each design model, the various models in Table 1 will be qualitatively compared to highlight the difference among them. It is worth noting that both models, the ACI 318-19 [174] and the CSA-A23.3-14 [175], are not considering the reinforcement ratio (ρ) and the ($\frac{a_{s}}{d}$) effects on concrete shear capacity. Similarly, in JSCE-1997 [176], the ($\frac{a_{s}}{d}$) effect is not counted. In the literature, a strong relationship between the shear capacity and varying (ρ) and ($\frac{a_{s}}{d}$) has been reported [141,142,166,169,170,171,172,178,179,180,181,182]. Hence, the exclusion of these variables is expected to result in inconsistent predictions. On the other hand, AASHTO LRFD-2012 [173] and the proposed equation by Xu et al. [177] have considered these effects. One source of inaccuracy in Xu et al.’s [177] model could be that the proposed equation predicts zero shear strength for concrete sections without flexural reinforcement. However, this is not a major problem since reinforcement exists in practical life applications.

As can be seen in Table 2, the replacement level of SCM with OPC is ranging between 20% to 70%. The concrete shear strength models of ACI 318-19 [174], CSA-A23.3-14 [175], and JSCE-1997 [176] have shown very conservative predictions with average experimental to predicted shear capacities ($V_{\exp}/V_{\mathrm{pred}}$) of 1.47 ± 0.42, 1.45 ± 0.38, and 1.51 ± 0.4, respectively. The proposed model of Xu et al. [177] also reported conservative predictions but with higher accuracy, where $V_{\exp}/V_{\mathrm{pred}}=$ 1.2 ± 0.22. In addition, Xu et al.’s [177] model recorded the least coefficient of variation, COV% = 18.61, which indicates better consistency compared to other models. On the contrary, AASHTO LRFD-2012 [173] over-estimated the predictions with $V_{\exp}/V_{\mathrm{pred}}=$ 0.95 ± 0.41 and COV% of 42.78. However, for those beams with 20% to 30% of FA, the AASHTO LRFD-2012 [173] has shown under-estimated predictions.

From the data in Table 3, it is apparent that RCA% is ranging from 25% to 100%. As the above observations, Xu et al.’s [177] model revealed the most accurate predictions with $V_{\exp}/V_{\mathrm{pred}}=$ 1.11 ± 0.29 and better consistency than the rest of the codes, with COV% of 26.34. On the other hand, AASHTO LRFD-2012 [173] demonstrated the least accurate predictions with $V_{\exp}/V_{\mathrm{pred}}=\;$1.03 ± 0.65 and COV% of 62.53. The predictions of AASHTO LRFD-2012 [173] appeared to be conservative for ($\frac{a_{s}}{d}$) less than 2.7, whereas the predictions were over-estimated for ($\frac{a_{s}}{d}$) greater than 2.7. The results, as shown in Table 3, indicate that the CSA-A23.3-14 [175] revealed the most conservative and high variability in predictions, with $V_{\exp}/V_{\mathrm{pred}}$ and COV% of 1.90 ± 0.83 and 43.95, respectively. Furthermore, both the JSCE-1997 [176] and ACI 318-19 [174] predictions in Table 3 were found to be consistent with those in Table 2, with $V_{\exp}/V_{\mathrm{pred}}$ of 1.58 ± 0.63 and 1.45 ± 0.50, and COV% of 40.20 and 34.62, respectively. As expected, the high variability in predictions accords well with our earlier qualitative comparison of the presented models in Table 1, which might be due to the exclusion of important factors such as ρ and $\frac{a_{s}}{d}$.

## 4. Conclusions

In this review, the sustainability benefits of GC were discussed first, then its mechanical properties when incorporating GGBFS, FA, SF, or MK as a partially replacing material for cement were analyzed at different replacement levels, different ages, and different W/b ratios in terms of compressive strength. The durability properties of GC were discussed under different environmental exposures. Following this, the structural behavior of GC in large-scale RC beams was analyzed and their concrete shear capacities were compared analytically to the available design codes, such as JSCE-1997 [176], ACI 318- 2019 [174], AASHTO LRFD-2012 [173], and CSA-A23.3-2014 [175], and a proposed equation by Xu et al. [177]. Based on the above review, the main outcomes are summarized as follow:

- The SF and MK were very effective in gaining higher early strength than the control mix with 100% OPC.
- At 90 days, the GGBFS concrete reported higher compressive strength than 40 MPa, except for concrete with 80% of GGBFS at W/b of 0.5.
- The analysis revealed that the 28-day strength of 20 to 35 MPa was achieved when the W/b range of 0.5 to 0.6 or 0.24 to 0.45 is implemented with a replacement level of FA of 10% to 30% or 40% to 60%, respectively.
- Higher strength grades (at 28 days) of the range 40 to 60 MPa and 60 to 80 MPa can be achieved when W/b ranges are 0.27 to 0.4 and 0.24 to 0.36 and when the replacement levels of FA are 10% to 55% and 10% to 40%, respectively.
- At the age of 28 days, high strength grades of the range 40 to 60 MPa and 60 to 90 MPa were achieved when W/b is of the range 0.35 to 0.5 and 0.26 to 0.4 and when the replacement ratio of SF is ranging from 5% to 20% and 5% to 25%, respectively.
- For concrete with MK, the 28-day strength of 60 to 80 MPa was achieved at W/b of 0.3 to 0.36 and with a replacement level in the range of 10% to 20%. At lower W/b of 0.27 to 0.33, the strength range of 80 to 100 MPa was achieved at replacement levels of 5% to 15%, respectively.
- At elevated temperatures higher than 400 °C, the concrete mixes with either GGBFS, FA, or SF demonstrated a sharp reduction in compressive strength.
- The sorptivity in pozzolanic cement pastes is remarkably lower than that in Portland cement paste.
- The long-term resistance to sulfate attack of concrete that combines GGBFS and FA was observed to be superior to the CC mix and high-volume FA concrete mix. Also, the former mix experienced less change in weight.
- The carbonation depth was shown to increase with the increased content of SCMs, and regardless of SCM type in concrete, the carbonation depth was higher than that of the control mix (with no SCM).
- The incorporation of silica nanoparticles (SNPs) could result in a significant reduction in the carbonation depth and the sulfate attack.
- Although the RCA concrete had a more permeable structure than the NCA concrete, the incorporation of FA, GGBFS, or SF can lead to a significant enhancement in the chloride ion penetration resistance.
- The concrete shear strength models of JSCE-1997 [176], ACI 318-19 [174], and CSA-A23.3-14 [175] have shown very conservative predictions for concrete beams with FA or RCA, whereas predictions were over-estimated by AASHTO LRFD-2012 [173].
- Among all models, the model of Xu et al. [177] revealed the most accurate predictions with $V_{\exp}/V_{\mathrm{pred}}=$ 1.2 ± 0.22 and 1.11 ± 0.29 for beams with FA or RCA, respectively.

Continued efforts are needed to determine the stress-strain behavior of GC to account for the required design considerations. In addition, further research could also be conducted to determine the GC behavior in large-scale specimens such as beams and slabs under shear and flexure, to develop an understanding of how the combination of GC, bars, and stirrups can create a system that is functional and safe. Another important aspect of research that might produce striking findings is to investigate the bond efficiency of GC with the conventional steel or with fiber-reinforced polymer (FRP) bars. On a wider level, there is a need for a holistic, detailed, and accurate social, economic, and environmental sustainability analysis of GC that considers all stages of GC product from cradle to grave. Finally, we believe that our research will serve as a base for analyzing other types of substitutes in GC, such as agricultural and municipal wastes.

## Figures and Tables

**Table 1:** Summary of code provisions and simplified shear design methods.

Code/Researcher	Equations
JSCE-1997	$V_{c}=\beta_{d}\beta_{p}\beta_{n}f_{\mathrm{vcd}}b_{w}d/\gamma_{b}$ $f_{\mathrm{vcd}}=0.2\sqrt[{3}]{f\prime c}\;,\;where\;f_{\mathrm{vcd}}\leq 0.72\;$(N/mm^2^) $\beta_{d}=\sqrt[{4}]{1000/d}$ $\beta_{p}=\sqrt[{3}]{100\rho }$ $\beta_{n}=1$ $\gamma_{b}=member\;factor\;taken\;as\;1.10$
ACI 318-19	$V_{c}=0.17\lambda \sqrt{f^{\prime }c}\;b_{w}d$
AASHTO LRFD-2012	$V_{c}=0.083\beta_{1}\lambda \sqrt{f^{\prime }c}\;b_{v}d_{v}$ $\beta_{1}=\frac{4.8}{1+750\varepsilon_{s}}$, $\varepsilon_{s}=\frac{\frac{M_{u}}{d_{v}}+V_{u}}{E_{s}A_{s}}$
CSA-A23.3-14	$V_{c}=\Phi_{c}{\;\beta }_{2}\lambda \sqrt{f^{\prime }c}\;b_{w}d_{v}$ $\beta_{2}=\frac{230}{1000+s_{\mathrm{ze}}}\;$, $s_{\mathrm{ze}}=\frac{35d_{v}}{15+a_{g}}$
(Xu et al. 2012)	$V_{c}=\frac{1.018}{\sqrt{d}}{\left(\frac{d}{a_{s}}\right)}^{\frac{1}{3}}\rho^{\frac{1}{6}}{\left(1-\sqrt{\rho }\right)}^{\frac{2}{3}}\left(0.0255f^{\prime }c+1.24\right)b_{w}d$

$f^{\prime }c=$ Concrete compressive strength, $b_{w}=$ beam width, d= beam effective depth, λ=1, $\beta_{1}=$ factor indicating the ability of diagonally cracked concrete to transmit tension and shear, $\varepsilon_{s}=$ the longitudinal tensile strain at the centroid of the tension reinforcement, $M_{u}=$ the factored moment, which should not be taken less than $V_{u}d_{v}$, $V_{u}=$ the factored shear force, $E_{s}=$ the young’s modulus, $A_{s}=$ the tensile reinforcement area, $b_{v}=$ effective web width taken as the minimum web width between the resultants of tensile and compressive forces due to flexure, $d_{v}=$ effective shear depth. It need not be less than the greater of 0.9d or 0.72 times beam height (h), $\Phi_{c}=$ the resistance factor for concrete. Selected as 1 in this study, $s_{\mathrm{ze}}$ is a crack spacing parameter, $a_{g}=$ maximum aggregate size, $a_{s}=$ shear span length of the beam.

**Table 2:** Experimental and predicted shear capacities for reinforced concrete beams with partial SCM%.

Source	SCM%	RCA%	ρ	$b_{w}\;(\mathrm{mm})$	*h* (mm)	*d* (mm)	$a_{g}\;(\mathrm{mm})$	$f^{\prime }c\;(\mathrm{MPa})$	$a_{s}/d$	Experimental $\mathrm{Shear},\;V_{c}\;(\mathrm{kN})$	$V_{exp}/V_{pred}$				
											JSCE-1997	ACI 318-19	AASHTO LRFD 2012	CSA-A23.3-14	Xu et al. [177]
Arezoumandi et al. [181]	50 (FA)	-	0.0126	305	457	396	19	30.7	3.0	127	1.36	1.12	0.70	1.16	1.04
	50 (FA)		0.0126	305	457	396	19	20.7	3.0	134.1	1.63	1.44	0.92	1.49	1.26
	50 (FA)		0.0199	305	457	375	19	30.7	3.2	163.9	1.56	1.52	0.91	1.56	1.33
	50 (FA)		0.0199	305	457	375	19	20.7	3.2	133.7	1.46	1.51	0.86	1.55	1.25
	50 (FA)		0.0266	305	457	375	19	30.7	3.2	164.8	1.43	1.53	0.85	1.57	1.30
	50 (FA)		0.0266	305	457	375	19	20.7	3.2	163.7	1.62	1.85	1.03	1.90	1.48
Arezoumandi et al. [182]	70 (FA)	-	0.0157	305	457	396	19	22	3.0	140.7	1.56	1.46	0.88	1.52	1.26
	70 (FA)		0.0199	305	457	375	19	22	3.2	131.9	1.41	1.45	0.82	1.48	1.21
	70 (FA)		0.0266	305	457	375	19	22	3.2	170.9	1.66	1.87	1.05	1.92	1.52
	70 (FA)		0.0266	305	457	375	19	21.6	3.2	162.9	1.59	1.80	1.00	1.85	1.45
	70 (FA)		0.0252	305	457	396	19	30.7	3.0	134.3	1.14	1.18	0.63	1.23	1.02
	70 (FA)		0.0252	305	457	396	19	20.7	3.0	122.8	1.19	1.31	0.69	1.37	1.07
	70 (FA)		0.0266	305	457	375	19	30.7	3.2	150.4	1.30	1.40	0.76	1.43	1.19
	70 (FA)		0.0266	305	457	375	19	20.7	3.2	168.1	1.66	1.90	1.06	1.95	1.52
	70 (FA)		0.0266	305	457	375	19	30.7	3.2	162.4	1.41	1.51	0.84	1.54	1.28
	70 (FA)		0.0266	305	457	375	19	20.7	3.2	201.5	1.99	2.28	1.34	2.33	1.82
Alghazali and Myers [142]	50 (FA)	-	0.0157	305	457	396	25	53.5	3.1	149.2	1.29	0.99	0.61	0.99	0.93
	50 (FA)		0.0199	305	457	375	25	53.5	3.3	143.8	1.19	1.01	0.58	0.99	0.91
	50 (FA)		0.0266	305	457	375	25	53.5	3.3	144	1.09	1.01	0.55	0.99	0.89
	60 (FA)		0.0157	305	457	396	25	45.9	3.1	142.5	1.24	1.02	0.62	1.02	0.96
	60 (FA)		0.0199	305	457	375	25	45.9	3.3	175.7	1.47	1.33	0.82	1.31	1.21
	60 (FA)		0.0266	305	457	375	25	45.9	3.3	150.6	1.14	1.14	0.62	1.12	1.00
	70 (FA)		0.0157	305	457	396	25	52.9	3.1	146.6	1.26	0.98	0.60	0.98	0.92
	70 (FA)		0.0199	305	457	375	25	52.9	3.3	162.2	1.35	1.15	0.69	1.13	1.04
	70 (FA)		0.0266	305	457	375	25	52.9	3.3	154.7	1.17	1.09	0.60	1.07	0.96
Sadati et al. [178]	50 (FA)	50	0.0126	305	460	396	25	30.8	3.0	120.5	1.29	1.06	0.65	1.05	0.99
	50 (FA)	50	0.0199	305	460	375	25	30.8	3.2	140.8	1.34	1.30	0.75	1.28	1.15
	50 (FA)	50	0.0266	305	460	375	25	30.8	3.2	136.3	1.18	1.26	0.68	1.24	1.07
Lisantono et al. [182]	50 (FA)	-	0.0106	150	260	214	25	15.3	3.5	57.3	2.64	2.68	2.24	2.36	1.73
	60 (FA)		0.0947	150	260	214	25	13.7	3.5	48.9	1.13	2.42	1.13	2.12	1.25
	70 (FA)		0.0947	150	260	214	25	11.7	3.5	41.9	1.02	2.24	1.03	1.97	1.11
Sunayana and Barai [141]	20 (FA)	100	0.0038	200	300	267	20	47.26	2.6	82.15	2.33	1.32	1.86	1.23	1.16
	30 (FA)	100	0.0038	200	300	267	20	45.55	2.6	81.8	2.35	1.34	1.88	1.25	1.18
	20 (FA)	100	0.0074	200	300	267	20	46.11	2.6	101.72	2.33	1.65	1.73	1.55	1.33
	30 (FA)	100	0.0074	200	300	267	20	47.6	2.6	87.49	1.98	1.40	1.35	1.31	1.13
Mean	-	-	-	-	-	-	-	-	-	-	1.51	1.47	0.95	1.45	1.20
SD	-	-	-	-	-	-	-	-	-	-	0.40	0.42	0.41	0.38	0.22
COV%	-	-	-	-	-	-	-	-	-	-	26.35	28.72	42.78	26.30	18.61

**Table 3:** Experimental and predicted shear capacities for reinforced concrete beams with partial or total RCA%.

Source	RCA%	ρ	$b_{w}\;(\mathrm{mm})$	*h* (mm)	*d* (mm)	$a_{g}\;(\mathrm{mm})$	$f^{\prime }c\;(\mathrm{MPa})$	$a_{s}/d$	Experimental $\mathrm{Shear},\;V_{c}\;(\mathrm{kN})$	$V_{exp}/V_{pred}$				
										JSCE-1997	ACI 318-19	AASHTO LRFD 2012	CSA-A23.3-14	Xu et al. [177]
Han et al. [166]	100	0.011	170	300	270	25	39.6	1.5	83.5	2.05	1.70	1.40	1.96	1.08
-	100	0.011	170	300	270	25	30.6	2	65.2	1.74	1.51	1.11	1.53	1.03
-	100	0.011	170	300	270	25	32.6	2	60.6	1.59	1.36	0.97	1.42	0.94
-	100	0.011	170	300	270	25	31.2	3	42.7	1.13	0.98	0.62	1.00	0.77
-	100	0.011	170	300	270	25	31.9	4	31.7	0.84	0.72	0.42	0.74	0.62
Belen and Fernando [179]	50	0.030	200	350	303	25	39.7	3.3	90.6	1.24	1.40	0.76	1.64	1.09
Etxeberria et al. [169]	25	0.030	200	350	303	25	42.4	3.3	104	1.39	1.55	0.88	1.89	1.21
-	50	0.030	200	350	303	25	41.3	3.3	89	1.20	1.34	0.73	1.62	1.05
-	100	0.030	200	350	303	25	39.8	3.3	84	1.15	1.29	0.70	1.52	1.01
Ji et al. [179]	100	0.012	170	300	270	20	39.7	2.2	60	1.45	1.22	0.85	1.61	0.87
Fathifazl et al. [170]	63.5	0.010	200	375	300	19	41.6	1.5	186.7	3.60	2.84	3.24	4.06	1.91
-	63.5	0.016	200	375	300	19	41.6	2	169.5	2.85	2.58	2.21	3.69	1.82
-	63.5	0.014	200	375	309	19	41.6	2.7	103.9	1.77	1.53	1.09	2.21	1.23
-	63.5	0.026	200	375	305	19	41.6	4	83.2	1.17	1.24	0.69	1.79	1.06
-	74.3	0.010	200	375	300	19	49.1	1.5	195.3	3.62	2.73	3.21	4.25	1.85
-	74.3	0.016	200	375	300	19	49.1	2	179	2.90	2.50	2.21	3.89	1.77
-	74.3	0.026	200	375	305	19	49.1	4	105.6	1.43	1.45	0.86	2.27	1.24
-	63.5	0.013	200	250	201	19	41.6	2.7	89.3	2.15	2.03	1.69	2.67	1.32
-	63.5	0.014	200	375	309	19	41.6	2.6	103.9	1.77	1.53	1.09	2.21	1.21
-	63.5	0.018	200	450	381	19	41.6	2.7	99.5	1.35	1.19	0.72	1.81	1.03
-	63.5	0.017	200	550	476	19	41.6	2.7	104.6	1.22	1.00	0.58	1.63	0.98
-	74.3	0.020	200	250	201	19	49.1	2.7	122.6	2.48	2.56	2.03	3.67	1.59
-	74.3	0.018	200	450	381	19	49.1	2.7	111.7	1.46	1.23	0.77	2.04	1.07
-	74.3	0.017	200	550	476	19	49.1	2.7	119.6	1.34	1.05	0.64	1.87	1.03
Knaack and Kurama [171]	50	0.0134	150	230	200	19	41.8	3.8	44	1.41	1.33	0.92	1.76	0.97
-	50	0.0134	150	230	200	19	41.8	3.8	39.1	1.25	1.19	0.78	1.57	0.86
-	50	0.0134	150	230	200	19	37.4	3.8	43.7	1.45	1.40	0.96	1.75	1.01
-	50	0.0134	150	230	200	19	37.4	3.8	41.2	1.37	1.32	0.89	1.65	0.96
-	100	0.0134	150	230	200	19	39.1	3.8	36.4	1.19	1.14	0.74	1.46	0.83
-	100	0.0134	150	230	200	19	39.1	3.8	38	1.24	1.19	0.78	1.52	0.86
-	100	0.0134	150	230	200	19	39.2	3.8	39.9	1.31	1.25	0.83	1.60	0.91
-	100	0.0134	150	230	200	19	39.2	3.8	36.1	1.18	1.13	0.73	1.45	0.82
Arezoumandi et al. [172]	100	0.0125	305	460	400	25	30	3.1	114.8	1.23	1.01	0.61	1.10	0.96
-	100	0.0199	305	460	375	25	30	3.25	143.2	1.38	1.34	0.78	1.45	1.18
-	100	0.0266	305	460	375	25	30	3.25	131.4	1.15	1.23	0.65	1.33	1.05
-	100	0.0125	305	460	400	25	34.1	3.1	113	1.16	0.93	0.57	1.09	0.89
-	100	0.0199	305	460	375	25	34.1	3.25	124.1	1.14	1.09	0.61	1.25	0.97
-	100	0.0266	305	460	375	25	34.1	3.25	140.3	1.18	1.24	0.66	1.42	1.07
Sadati et al. [178]	50	0.013	305	460	396	25	32	3.0	117.4	1.24	1.01	0.62	1.14	0.95
-	50	0.020	305	460	375	25	32	3.2	151.2	1.42	1.37	0.81	1.53	1.21
-	50	0.027	305	460	375	25	32	3.2	171.7	1.47	1.56	0.88	1.73	1.33
Mean	-	-	-	-	-	-	-	-	-	1.58	1.45	1.03	1.90	1.11
SD	-	-	-	-	-	-	-	-	-	0.63	0.50	0.65	0.83	0.29
COV%	-	-	-	-	-	-	-	-	-	40.20	34.62	62.53	43.95	26.34

## Data Availability

Data is contained within the article or Supplementary Material. The data presented in this study are available in Al-Hamrani, A.; Kucukvar, M.; Alnahhal, W.; Mahdi Saad, E.; Onat, N.C. Green Concrete for a Circular Economy: A Review on Sustainability, Durability, and Structural Properties.

## Supplementary Materials

The following are available in an excel sheet online at https://www.mdpi.com/1996-1944/14/2/351/s1, Table S1: GGBFS concrete, Table S2: FA concrete, Table S3: SF concrete, Table S4: MK concrete.

## Abbreviations

CE	Circular economy
PCC	Portland cement concrete
C&D	Construction and demolition
GC	Green concrete
NCA	Natural coarse aggregates
RCA	Recycled coarse aggregates
OPC	ordinary Portland cement
SCM	Supplementary cementitious material
GGBFS	Ground granulated blast furnace slag
FA	Fly ash
SF	Silica fume
MK	Metakaolin
RHA	Rice husk ash
LCA	Life cycle assessment
CC	Conventional concrete
EAF	Electric arc furnace
GWP	Global warming potential
C-S-H	Calcium silicate hydrate
RC	Reinforced concrete
GGFAC	Concrete with a combination of GGBFS and FA
VA	Volcanic ash
DS	Drying shrinkage
SNPs	Silica nanoparticles
HVFA	High-volume FA
